# Suppression of Starvation-Induced Autophagy by Recombinant Toxic Shock Syndrome Toxin-1 in Epithelial Cells

**DOI:** 10.1371/journal.pone.0113018

**Published:** 2014-11-17

**Authors:** Krisana Asano, Yoshiya Asano, Hisaya K. Ono, Akio Nakane

**Affiliations:** 1 Department of Microbiology and Immunology, Hirosaki University Graduate School of Medicine, Hirosaki, Aomori, Japan; 2 Department of Neuroanatomy, Cell Biology and Histology, Hirosaki University Graduate School of Medicine, Hirosaki, Aomori, Japan; The University of Hong Kong, Hong Kong

## Abstract

Toxic shock syndrome toxin-1 (TSST-1), a superantigen produced from *Staphylococcus aureus*, has been reported to bind directly to unknown receptor(s) and penetrate into non-immune cells but its function is unclear. In this study, we demonstrated that recombinant TSST-1 suppresses autophagosomal accumulation in the autophagic-induced HeLa 229 cells. This suppression is shared by a superantigenic-deficient mutant of TSST-1 but not by staphylococcal enterotoxins, suggesting that autophagic suppression of TSST-1 is superantigenic-independent. Furthermore, we showed that TSST-1-producing *S. aureus* suppresses autophagy in the response of infected cells. Our data provides a novel function of TSST-1 in autophagic suppression which may contribute in staphylococcal persistence in host cells.

## Introduction

Autophagy is a fundamental cellular homeostatic mechanism that provides for bulk degradation of organelles and cytosolic proteins [1]. During autophagy, parts of cytoplasm and organelles are encapsulated into double membrane vacuoles called autophagosomes, which encounter the lysosomes to digest the sequestered recycling components for self-support [2]. Autophagy-mediated proteolysis plays a crucial role in survival, growth, proliferation and differentiation of eukaryotic cells [3], [4]. In addition, autophagy is involved in the defense against several pathogenic microorganisms [5]. It was previously postulated that some intracellular bacteria are targeted by autophagic degradation system [6]. They are sequestrated within autophagosomes, which ultimately deliver the microorganisms to lysosome to be eliminated. However, successful pathogens have evolved strategies to avoid autophagy, or to actively subvert its components, to promote their own replication [6], [7].

*Staphylococcus aureus* is an important human pathogen which causes a variety of infection ranging from superficial infections to more life-threatening diseases [8]. *S. aureus* has been classically considered as an extracellular pathogen but numerous studies have shown that *S. aureus* can invade cells and replicate intracellularly [9]. This bacterium is able to infect various types of nonprofessional phagocytic host cells such as keratinocytes, fibroblasts, endothelial and epithelial cells [10], [11]. One of the key features of *S. aureus* infection is the production of series of virulence factors, including secreted enzymes and toxins whose expression is regulated by a set of global virulence regulators [12], [13]. Previous studies suggested a connection between autophagic response and *S. aureus* infection which occurs via the bacterial *agr*-virulence factor [14], [15], [16]. Pore-forming α-hemolysin, regulated by the *agr* is shown to participate in the activation of the autophagic pathway [15].

Toxic shock syndrome toxin-1 (TSST-1) is one of pyrogenic superantigens secreted by *S. aureus*. Potent effects of TSST-1 on host immune system have been largely elucidated [12]. This toxin directly crosslinks between the major histocompatibility complex class II molecule on antigen-presenting cell and T cell receptor bearing specific Vβ element. This binding subsequently leads to a massive proliferation of T cells and the uncontrolled release of proinflammatory cytokines [17], [18]. Previous studies have shown that TSST-1 binds to an uncharacterized receptor(s) on endothelial cells and epithelial cells [19], [20] and penetrate into epithelial cells [21]. In addition, immunization with recombinant and/or mutant TSST-1 protects mice against systemic *S. aureus* infection [22], [23]. Except for neutralization of superantigenic activity, these toxin-specific antibodies alter bacterial growth in the organs of mice. These data suggest another biological function of this toxin in the non-immune cells. Although the production of this toxin is under the control of several regulatory proteins, its expression is also partially regulated by the *agr* [24]. In this study, we investigated the effect of TSST-1 on autophagy in HeLa 229 cells. Our results suggest that TSST-1 suppresses autophagy. Furthermore, this suppression is superantigenic activity-independent.

## Materials and Methods

### Bacterial strains and growth conditions

*S. aureus* 834 wild type (WT), a clinical septic isolate that produces TSST-1 [25], and its derivative TSST-1-deficient mutant (Δ*tst*) were cultured at 37°C in tryptic soy broth (BD Bioscience, Sparks, MD) or tryptic soy agar for 16 h. The bacterial cells were collected, suspended in phosphate-buffered saline (PBS) and adjusted spectrophotometrically at 550 nm.

### Cell line and cell culture condition

Human cervical carcinoma HeLa 229 cells were cultured in Eagle’s minimal essential medium (MEM, Nissui Pharmaceutical Co., Tokyo, Japan), supplemented with 10% fetal bovine serum (JRH Biosciences, Lenexa, KS), 0.03% of L-glutamine (Wako Pure Chemical Industries, Ltd., Osaka, Japan) and 1x non-essential amino acids (Invitrogen, Carlsbad, CA) at 37°C and 5% CO_2_.

### Expression of green fluorescent protein (GFP) linked with microtubule-associated protein 1-light chain 3 (LC3) as autophagy marker in HeLa 229 cells

Human LC3B coding gene was amplified from cDNA obtained from human epithelial kidney HEK293 cells with primer LC3BF and LC3BR (5′-GAATTCATGCCGTCGGAGAAGACCTT-3′ and 5′-GGTACCTTACACTGACAATTTCATCCCG-3′, respectively). LC3B coding gene was inserted into pEGFP-C2 plasmid (BD Biosciences Clontech, Palo Alto, CA) to construct pEGFP-hLC3 plasmid. HeLa 229 cells were seeded in 24-well plates and transfected with pEGFP-hLC3 plasmid using Lipofectamine 2000 (Invitrogen) according to manufacturer’s instructions. Cells transfection with pEGFP-C2 plasmid was used as mock control. After 24 h of transfection, green fluorescence was observed under confocal microscope (Nikon Eclipse C1si, Nikon, Tokyo, Japan).

### Preparation of recombinant TSST-1 (rTSST-1), mutant TSST-1 (mTSST-1) and staphylococcal enterotoxins (SEs)

rTSST-1, mTSST-1, SEA, SEB and SEC were prepared as described in the File S1. To eliminate the effect of lipopolysaccharides (LPS), the contaminated LPS in the purified proteins was removed by Proteospin endotoxin removal kit (Norgenbiotek, ON, Canada) according to manufacturer’s instructions.

### Autophagic induction, lysosomal staining and immunostaining

Autophagy in HeLa 229 cells was induced by nutrient-starvation or rapamycin treatment. For nutrient-starvation, HeLa 229 cells were washed and incubated with Krebs Ringer bicarbonate buffer pH 7.6 (KRB; 118.5 mM NaCl, 4.47 mM KCl, 1.18 mM KH_2_PO_4_, 23.4 mM NaHCO_3_, 6 mM glucose, 2.5 mM CaCl_2_, 1.18 mM MgSO_4_, and 6 mg/l phenol red) for 0–6 h. For rapamycin treatment, cells were incubated with 1 µM rapamycin [stock 1 mM in dimethyl sulfoxide (DMSO), Sigma Aldrich, St. Louis, MO] in MEM for 4 h. Lysosomal protease inhibitors, 10 µg/ml E64d (Peptide Institute, Inc., Osaka, Japan) and 10 µg/ml pepstatin A (Peptide Institute, Inc.) were used to inhibit the lysosomal turnover. For immunostaining, the cells were fixed with 4% paraformaldehyde (Wako), washed with PBS, and lysed with 50 µg/ml digitonin (Wako). After quenching in 50 mM NH_4_Cl, the cells were blocked in 2% (w/v) bovine serum albumin, 5% (v/v) normal goat serum in 20 mM Tris-HCl, 150 mM NaCl. Lysosomes were immunostained with anti-lysosomal-associated membrane protein 1 (LAMP1) antibody (Sigma Aldrich) and rhodamine-conjugated anti-rabbit immunoglobulin G (IgG) (MP Biomedicals, Irvine, CA), whereas autophagosomes were immunostained with anti-LC3 antibody (Sigma Aldrich) and fluorescein isothiocyanate (FITC)-conjugated anti-rabbit IgG (MP Biomedicals). Fluorescence signal was observed under confocal microscope.

### Electron microscopy

HeLa 229 cells were cultivated on sterilized glass slides and the autophagy was induced under nutrient-starvation condition with or without 10 µg/ml rTSST-1 in the presence of lysosomal protease inhibitors. At 4 h after induction, the cells were fixed with 4% paraformaldehyde, 1% glutaraldehyde (Wako) in PBS, and then post-fixed with 1% osmium tetroxide (Heraeus Chemicals, Port Elisabeth, South Africa) in 0.1 M phosphate buffer (pH 7.4). The samples were dehydrated through a graded series of ethanol (Wako) and propylene oxide (Wako) at room temperature, and embedded in Epon 812 resin (TAAB Laboratories Equipment Ltd., Berkshire, UK). They were then polymerized with the resin in gelatin capsules (No. 0; Eli Lilly Co., Indianapolis, IN) at 60°C for 48 h. After polymerization, the samples on glass slides were transferred to resin block. Ultra-thin sections (70–80 nm) were cut with a diamond knife, stained with Sato’s lead citrate [26] and uranyl acetate (Merck, Darmstadt, Germany), and observed under a transmission electron microscope JEM 1250 (JEOL Ltd., Tokyo, Japan) at 80 kV.

### SDS-PAGE and Western blotting

After induction of autophagy in the presence of rTSST-1, mTSST-1, SEA, SEB or SEC with and without lysosomal protease inhibitors, crude proteins from HeLa 229 cells were collected in lysis buffer [2% triton X-100 in PBS containing complete protease inhibitor cocktail (Roche Diagnostics, Mannheim, Germany)] and applied to 12.5% polyacrylamide gel. The proteins were transferred to polyvinylidene fluoride membrane (Immobilon-P, Millipore, Bedford, MA). The membrane was then blocked for 2 h with 5% skim milk in Tris-buffered saline (20 mM Tris-pH 7.5, 150 mM NaCl, 0.05% Tween 20), washed twice with Tris-buffered saline, and incubated with a primary antibody anti-LC3 (Sigma) or β-tubulin (Santa Cruz Biotechnology, Inc., CA). The signal was detected by peroxidase-conjugated anti-rabbit IgG (MP Biomedicals) and SuperSignal West Dura Extended Duration Substrate (Pierce Biotechnology Inc., Rockfored, IL). The intensity of LC3-II band was quantified using Image Lab software normalized with intensity of β-tubulin band. The amount of LC3-II was calculated relatively to the amount of LC3-II from autophagic induction condition with lysosomal protease inhibitors, which set to 1.

### *S. aureus* infection

*S. aureus* Δ*tst* was constructed from the WT (File S1). GFP-LC3-expressing HeLa 229 cells were infected with *S. aureus* 834 or Δ*tst* at multiplicity of infection of 100. After incubation for 45 min, the extracellular bacteria were eliminated with 100 µg/ml lysostaphin (Wako). At 6 h of infection, the cells were fixed, washed, lysed and blocked as described above. *S. aureus* cells were immunostained with anti-Staph. aureus antibody (ViroStat, Inc, Portland, ME) and rhodamine-conjugated anti-rabbit IgG (MP Biomedical). *S. aureus* cells and GFP-LC3 puncta were observed from under confocal microscope.

### Statistical analysis

Data were expressed as means ± standard deviations, and *P*<0.05 from student’s *t* test analysis was used to determine the significance of the differences.

## Results

### rTSST-1 suppresses autophagosome accumulation in nutrient-starved HeLa 229 cells

**Figure 1:**
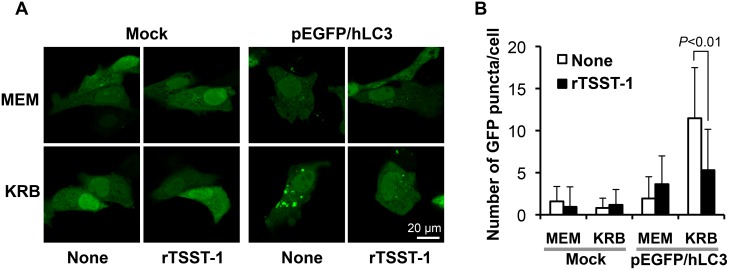
rTSST-1 suppresses autophagy in nutrient-starved HeLa 229 cells. HeLa 229 cells were transfected with pEGFP-hLC3 or pEGFP-C2 (Mock). Effect of TSST-1 was observed in nutrient-rich (MEM) and nutrient-starvation (KRB) condition by addition of 10 µg/ml rTSST-1. At 6 h, GFP-LC3 puncta were assessed under confocal microscope (A). GFP-LC3 puncta were counted from 100 cells of 3 independent-experiments (B).

To investigate the effect of TSST-1 on autophagy, LPS-free rTSST-1 was prepared and HeLa 229 cells were transfected with pEGFP-hLC3 plasmid. The effect of rTSST-1 on autophagy was then investigated in the GFP-LC3 expressing cells under both nutrient-rich (MEM) and nutrient-starvation (KRB) condition. As shown in Figure 1A–1B, the GFP-LC3 puncta in mock-transfected cells (transfection with pEGFP-C2 plasmid) was not observed in any conditions (MEM and KRB with and without rTSST-1). For the pEGFP-hLC3-transfected cells under nutrient-rich condition (MEM) in which autophagy was not induced, only small amount of GFP-LC3 puncta in these cells was observed. On the other hand, in the pEGFP-hLC3-transfected cells under nutrient-starvation condition (KRB) in which autophagy was induced, the average of GFP-LC3 puncta up to 11 per cell was found. Importantly, rTSST-1 did not significantly alter and/or induce the amount of GFP-LC3 puncta in the cells under nutrient-rich condition. In contrast, the amount of GFP-LC3 puncta in the autophagic-induced cells was significantly reduced by the addition of 10 µg/ml rTSST-1. The results indicate that rTSST-1 suppresses the autophagosomal accumulation in the autophagic-induced HeLa 229 cells.

### rTSST-1 does not enhance lysosomal fusion and autophagosomal degradation

**Figure 2:**
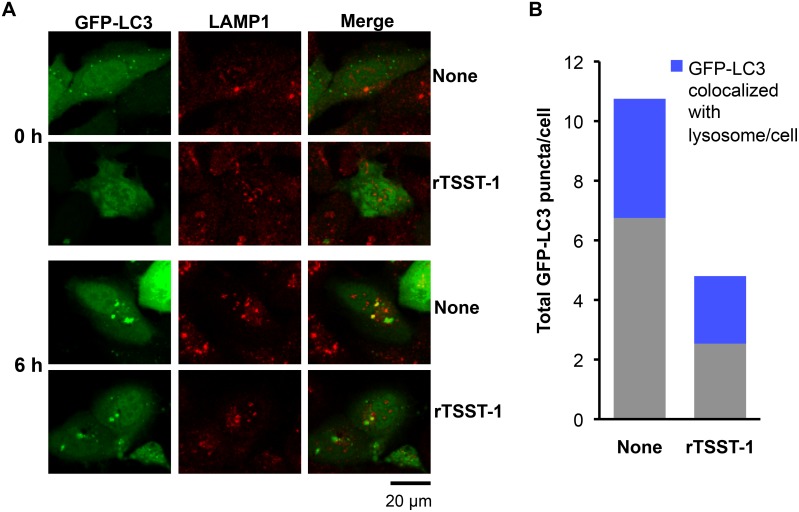
rTSST-1 does not enhance autophagosome and lysosome fusion. HeLa 229 cells were transfected with pEGFP-hLC3. Autophagy was induced under nutrient-starvation condition for 0 and 6 h with or without the addition of 10 µg/ml rTSST-1. Lysosomes were immunostained with LAMP1, lysosomes and GFP-LC3 puncta were observed under confocal microscope (A). GFP-LC3 puncta and overlapping between GFP-LC3 and lysosomes were counted from 100 cells of 2 independent-experiments (B).

**Figure 3:**
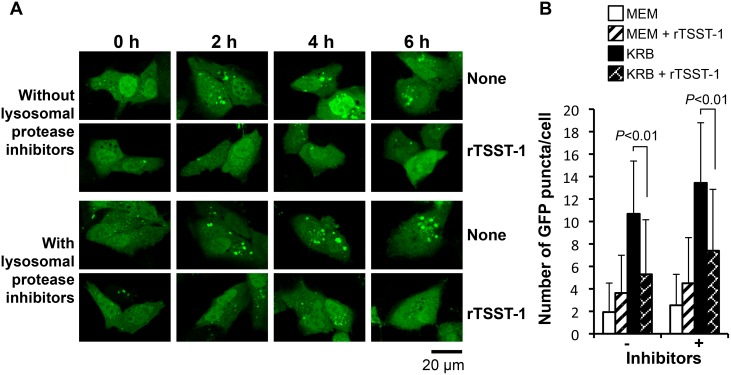
Lysosomal protease inhibitors fail to restore autophagosomes in TSST-1-treated cells. HeLa 229 cells were transfected with pEGFP/hLC3 and autophagy was induced under nutrient-starvation with or without the addition of 10 µg/ml rTSST-1 and lysosomal protease inhibitors. At the indicating time, the autophagosomal accumulation in the cells was observed by GFP puncta under confocal microscope (A). GFP-LC3 puncta at 4 h were counted from 100 cells of 2 independent-experiments (B).

**Figure 4:**
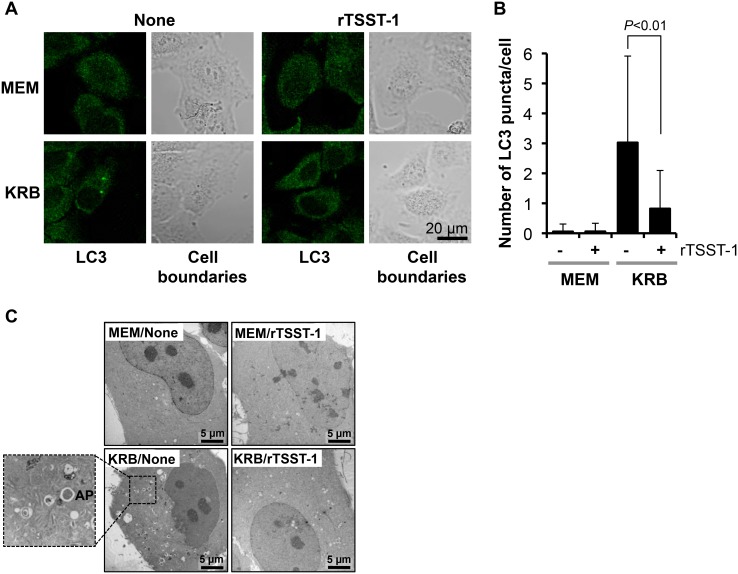
Suppression of autophagy by rTSST-1 is observed by immunostaining and electron microscopy. Autophagy in HeLa 229 cells was observed in nutrient-rich (MEM) or nutrient-starvation (KRB) condition containing lysosomal protease inhibitors with or without the addition of 10 µg/ml rTSST-1. At 4 h, the cells were fixed and washed. (A) Autophagosomes were stained with anti-LC3 antibody and rhodamine-conjugated anti-rabbit IgG, and then observed under confocal microscope. (B) LC3 puncta were counted from 100 cells of 3 independent-experiments. (C) Autophagosomes were observed under electron microscope. AP indicates autophagosome-like vacuole.

Autophagy is a dynamic process which comprises autophagosomal synthesis and autophagosomal degradation. In order to determine whether the autophagosome suppression by rTSST-1 is involved in lysosomal fusion process, lysosomes in the GFP-LC3-expressing HeLa 229 cells were immunostained with LAMP1 and the overlapping between GFP-LC3 and lysosome was observed. As shown in Figure 2A–2B, total amount of GFP-LC3 puncta was reduced by the addition of rTSST-1, correlating to the results obtained in Figure 1. However, the percent of lysosomes fused with GFP-LC3 puncta was not significantly changed or enhanced by the addition of rTSST-1. It was around 41–47% of total GFP-LC3 puncta per cell. Similar results were also obtained by staining acidic pH of lysosomes using LysoTracker Red (Figure S1). We further observed whether rTSST-1-dependent autophagosome suppression is involved in an enhancement of autophagosomal degradation. Autophagic flux was observed at various times of autophagic induction with or without the addition of the lysosomal protease inhibitors. As expected, the GFP-LC3 puncta which increased upon the time of autophagic induction was suppressed by rTSST-1 (Figure 3A–3B). Furthermore, the addition of lysosomal protease inhibitors failed to restore autophagosomes in the rTSST-1-treated cells. These results indicate that rTSST-1 does not enhance autophagosome-lysosome fusion and autophagosomal degradation. To avoid false interpretation that may occur by overexpression of GFP-LC3, the autophagic-suppressing activity of rTSST-1 was confirmed by immunostaining of LC3 and electron micrographs (Figure 4A–4C). The number of LC3 puncta from immunostaining and autophagosome-like vacuoles in electron micrographs was reduced by the addition of rTSST-1 to the nutrient-starved cells that supplemented with lysosomal protease inhibitors.

### rTSST-1 suppresses LC3-II accumulation in both KRB and rapamycin treatment

**Figure 5:**
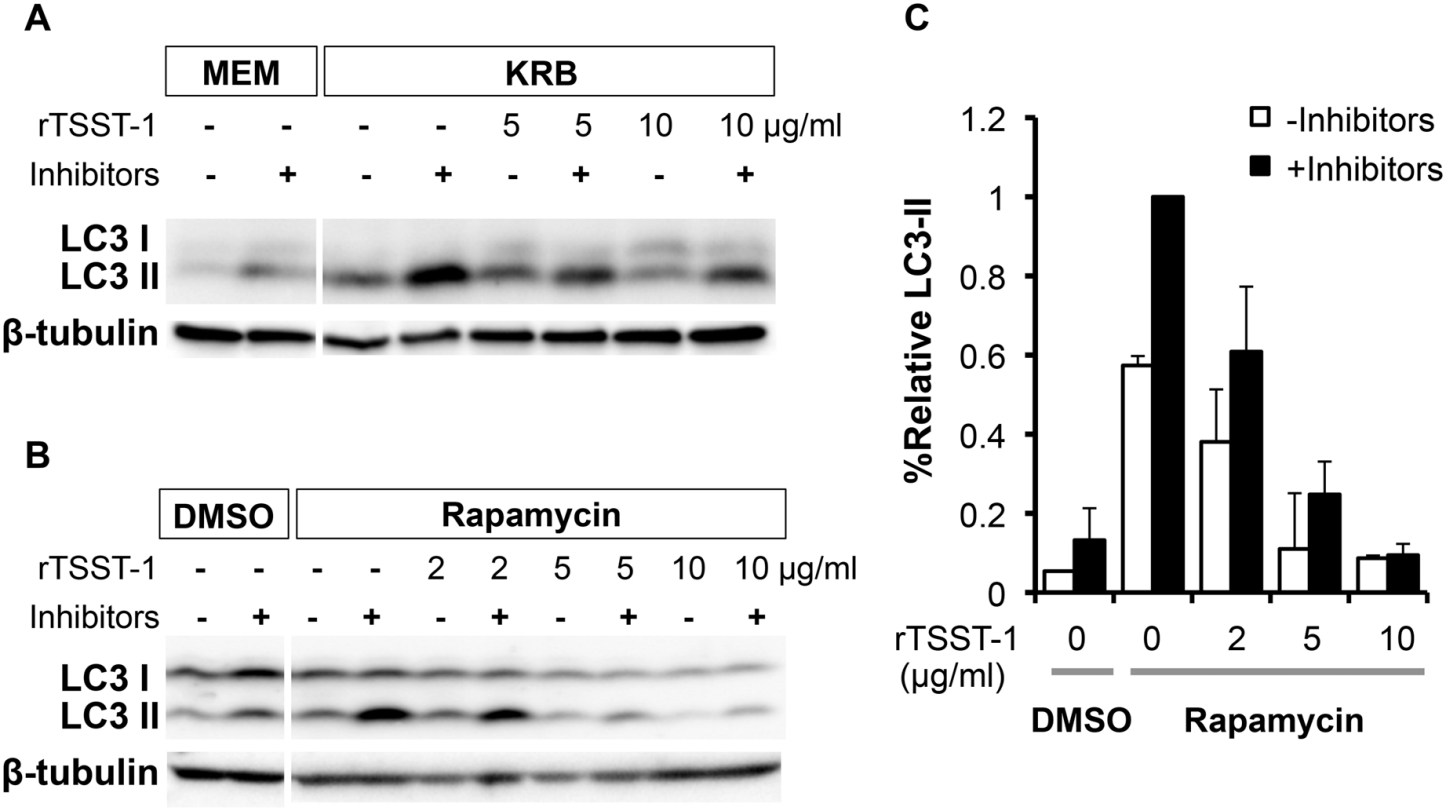
rTSST-1 suppresses LC3-II accumulation in the autophagy induced HeLa 229 cells. Autophagy in HeLa 229 cells was induced by nutrient-starvation (KRB) (A) or rapamycin (B and C) with or without the addition of lysosomal protease inhibitors and rTSST-1. Cells in MEM or DMSO were used as controls. At 4 h of induction, LC3-II was detected by Western blotting (A and B). (C) The intensity of LC3-II band was quantified by normalizing with the intensity of β-tubulin band. The amount of LC3-II was calculated relatively to that from autophagic induction condition with lysosomal protease inhibitors, which set to 1.

LC3-II accumulation is an important marker for autophagosome. We also examined the accumulation of LC3-II in the HeLa 229 cells by Western blotting using anti-LC3 antibody. As expected, the results in Figure 5A indicated that the amount of LC3-II in the nutrient-starved cells (KRB) was significantly higher than that of nutrient-rich condition (MEM). In addition, rTSST-1 suppressed the accumulation of LC3-II in these cells in a dose-dependent manner. A similar result was also found in the cells treated with rapamycin (Figure 5B). The amount of LC3-II in the cells treated with rapamycin was significantly higher than that of DMSO control and the amount of LC3-II in the rapamycin-treated cells was reduced by the addition of rTSST-1 in a dose-dependent manner (Figure 5C). A dose-dependent response of rTSST-1 analyzed by GFP-LC3 puncta formation in the nutrient-starved cells was also shown in Figure S2.

### TSST-1-producing *S. aureus* suppresses autophagy

**Figure 6:**
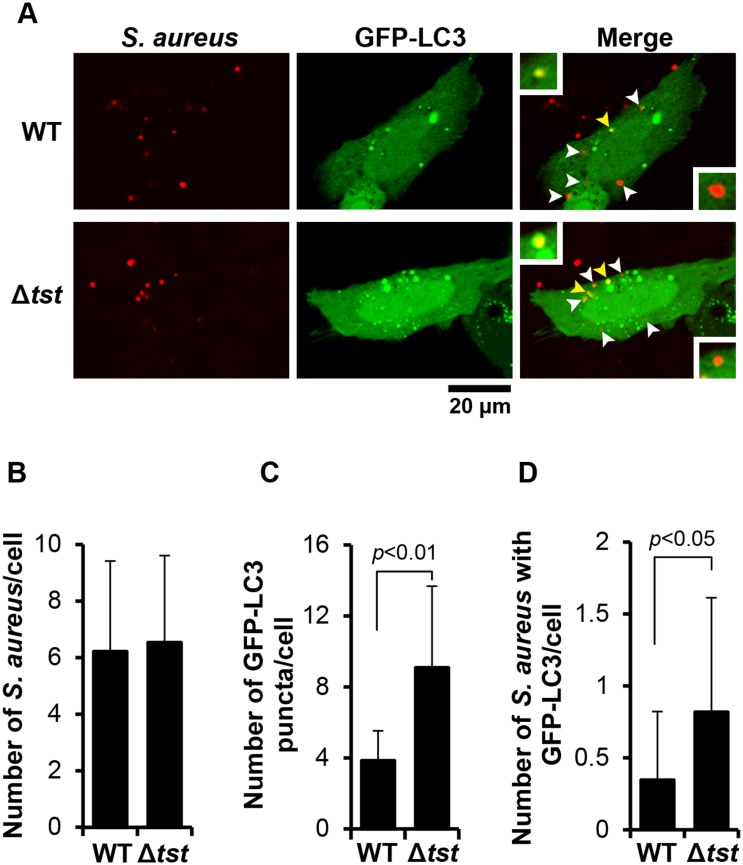
TSST-1-producing *S. aureus* suppresses autophagy. HeLa 229 cells were transfected with pEGFP-hLC3 plasmid and infected with *S. aureus* 834 or Δ*tst*. At 6 h of infection, *S. aureus* cells were immunostained as described in the Experimental procedure. GFP-LC3 and *S. aureus* cells were observed under confocal microscope (A). LC3-colocalized *S. aureus* spots are represented and indicated by upper inset and yellow arrowheads, respectively. LC3-free *S. aureus* spots are represented and indicated by lower inset and white arrowheads, respectively. GFP-LC3 puncta (B), *S. aureus* cells (C) and colocalization of *S. aureus* with GFP-LC3 (D) were analyzed from at least 100 cells of 3 independent-experiments.

To confirm whether TSST-1 produced by *S. aureus* suppresses autophagy, Δ*tst* was constructed and characterized (see File S1 and Figure S3–S7). After infecting the pEGFP-hLC3-transfected HeLa 229 cells with WT or Δ*tst* for 6 h, *S. aureus* cells were immunostained with anti-*S. aureus* antibody. The amount of GFP-LC3 puncta in the cells with an equivalent number of *S. aureus* was analyzed. As shown in the Figure 6A–6D, the number of *S. aureus* between WT and Δ*tst* in the selected cell was not significantly different but the GFP-LC3 puncta in the cells infected with Δ*tst* were higher than those with the WT. In addition, colocalization of Δ*tst* cells with GFP-puncta was also higher than that with the WT. The results suggest that the TSST-1-producing *S. aureus* suppresses autophagy in the response of infection.

The enhancement of GFP-LC3 puncta due to Δ*tst*-infection was not only found in HeLa 229 but also found in the human epithelial kidney HEK293 cells and human intestinal epithelial 407 cells (Figure S8). The effect of TSST-1 on autophagy suppression during *S. aureus* infection was confirmed by Western blotting. The results demonstrated that LC3-II accumulation in the Δ*tst*-infected cells was reduced by addition of rTSST-1 (Figure S9).

### Suppression of autophagy by TSST-1 does not depend on superantigenic activity

**Figure 7:**
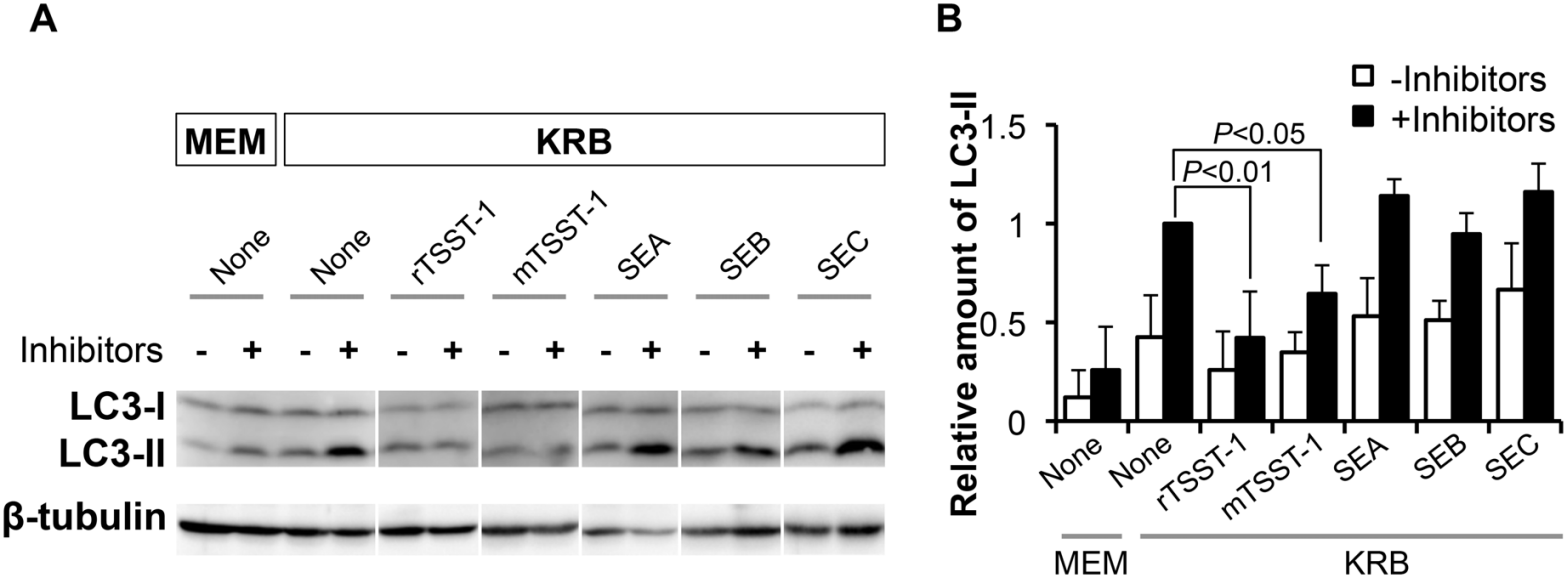
Suppression of LC3-II accumulation by rTSST-1 in the cells is superantigenic activity-independent. Autophagy in HeLa 229 cells was induced by nutrient-starvation (KRB) with or without lysosomal protease inhibitors and 10 µg/ml rTSST-1, mTSST-1, SEA, SEB or SEC. Cells in MEM were used control. At 4 h of induction, LC3-II was detected by Western blotting (A) and the intensity of LC3-II band was quantified (B) as described in Figure 5C. The data is provided as SD of at least 3-independent experiments.

In order to determine whether suppression of autophagosome depends on superantigenic activity of TSST-1, the accumulation of LC3-II in the nutrient-starved cells was observed by addition of mTSST-1 or SEs (SEA, SEB and SEC). mTSST-1 is a H135A mutant of rTSST-1 lacking of superantigenic activity, whereas the SEA, SEB and SEC are enterotoxins that exhibit superantigenic activity. The results in Figure 7A–7B demonstrated that the rTSST-1 and mTSST-1 had a similar autophagic-suppressing effect. In contrast, SEA, SEB and SEC did not suppress the accumulation of LC3-II in the autophagic induced cells. These results indicate that the autophagic suppression is specific to TSST-1 and does not depend on its superantigenic activity.

## Discussion

Besides well-clarified superantigenic activity of TSST-1 in immune cells, our results suggest a novel function of TSST-1 in epithelial cells that is participating in autophagy. We demonstrated that TSST-1 suppresses autophagy in the autophagic-induced HeLa 229 cells. Non-increasing of autophagosome-lysosome fusion and non-restoring of autophagy by the addition of lysosomal protease inhibitors suggested that rTSST-1 may inhibit autophagosomal synthesis rather than enhance autophagosome degradation. In addition, this autophagic suppression was similarly found in the cells induced with nutrient-starvation and rapamycin treatment, suggesting that TSST-1 may suppress canonical autophagy pathway. Although TSST-1 shares superantigenic activity with SEs, its primary sequence is shorter and has a homologous limitation. Unlike rTSST-1 and mTSST-1, SEs did not suppress autophagy. These results suggest that autophagic suppressing activity of TSST-1 does not depend on superantigenic activity. In contrast, it requires a specific structure of TSST-1 that does not share with SEs.

The reason of autophagic suppression by TSST-1 is still elusive. In the current report, we also presented evidence that not only purified rTSST-1 is able to suppress autophagy in the autophagic-induced cells but also the TSST-1-secreting *S. aureus* suppresses autophagy in the response of infection. Thus, autophagic suppression by TSST-1 might contribute in staphylococcal infection. *S. aureus* is known as a major human pathogen which can be carried by healthy persons [27]. To escape host immune response, *S. aureus* has an effective strategy of persistence on mucosal surface and hiding within the host cells. Previous studies demonstrated that superantigenic activity of TSST-1 can modulate immune response, leading to an immunosuppressive state [12], [18]. However, local production of TSST-1 may be insufficient to cause large-scale systemic immunosuppression. Thus, the local effect of TSST-1 at the colonization site might promote persistence of organism. Tuchscherr and coworkers demonstrated that *S. aureus* is able to invade and persist within non-phagocytic cells for several weeks after the infection [28]. To invade into the non-phagocytic cells, actin cytoskeleton reorganization regulated by integrin-linked kinase is required [29]. Our results indicated that entry of *S. aureus* into epithelia does not interfere with TSST-1 (Figure S3–S5). After entry into the host cells, *S. aureus* requires appropriate characteristics to survive intracellularly including not killing the host cells and resisting or non-activating intracellular host defenses. Although *S. aureus* has been shown to induce autophagy via cAMP down regulation [30], the effect of autophagy to intracellular *S. aureus* is still unclear. In the model of Schnaith and coworkers, *agr*-positive *S. aureus* localizes in autophagosome-like vesicles, where *S. aureus* replicates and subsequently escapes into the cytoplasm, to promote host cell death [14]. On the other hand, Mauthe and coworkers found that *S. aureus* cells were entrapped in autophagosome-like vesicles which then are targeted for lysosomal degradation [16]. In fact, to survive in the host cells without induction of host cell death, *S. aureus* needs to down-regulate autophagy. It has been shown that percent persistence of TSST-1 producing strains is higher than non-producing strains [31]. Thus suppression of autophagy by TSST-1 might be an alternative strategy of *S. aureus* for persistence in the host cells. To demonstrate this idea, the intracellular bacterial number as well as the viability of *S. aureus*-infected cells should be investigated and compared between *S. aureus* WT and Δ*tst* in further experiments. Although these intracellular persisting behaviors remained to be compared, the data in this study provides a novel function of TSST-1 participating in autophagic suppression.

## Supporting Information

Figure S1 **rTSST-1 does not enhance autophagosome and lysosome fusion.**
(TIF)

Figure S2 **rTSST-1 suppresses GFP-LC3 puncta formation in the nutrient-starved HeLa 229 cells in a dose-dependent manner.**
(TIF)

Figure S3 **Construction of TSST-1 deficient mutant.**
(TIF)

Figure S4 **Growth of Δ** ***tst*** **is comparable with that of the WT.**
(TIF)

Figure S5 **Effect of TSST-1 on adhesion assay.**
(TIF)

Figure S6 **Effect of TSST-1 on invasion assay at MOI 10 and 100.**
(TIF)

Figure S7 **Effect of TSST-1 on invasion assay at MOI 100 in the presence of rTSST-1.**
(TIF)

Figure S8 **TSST-1-producing** ***S. aureus*** **suppresses autophagy in HEK293 and 407 cells.**
(TIF)

Figure S9 **LC3-II accumulation in the Δ** ***tst*** **-infected cells was reduced by addition of rTSST-1.**
(TIF)

File S1 **Supporting information describing methodologies and results of Lysotracker-stained lysosomes,** ***tst*** **mutant construction and effect of TSST-1 on adhesion and invasion assay of** ***S. aureus*** **.**
(DOCX)
