# Supplementary figures and images for: Suppression of Starvation-Induced Autophagy by Recombinant Toxic Shock Syndrome Toxin-1 in Epithelial Cells
Source: PLoS One. 2014 Nov 17;9(11):e113018. doi: 10.1371/journal.pone.0113018 (PMC4234639; doi:10.1371/journal.pone.0113018)

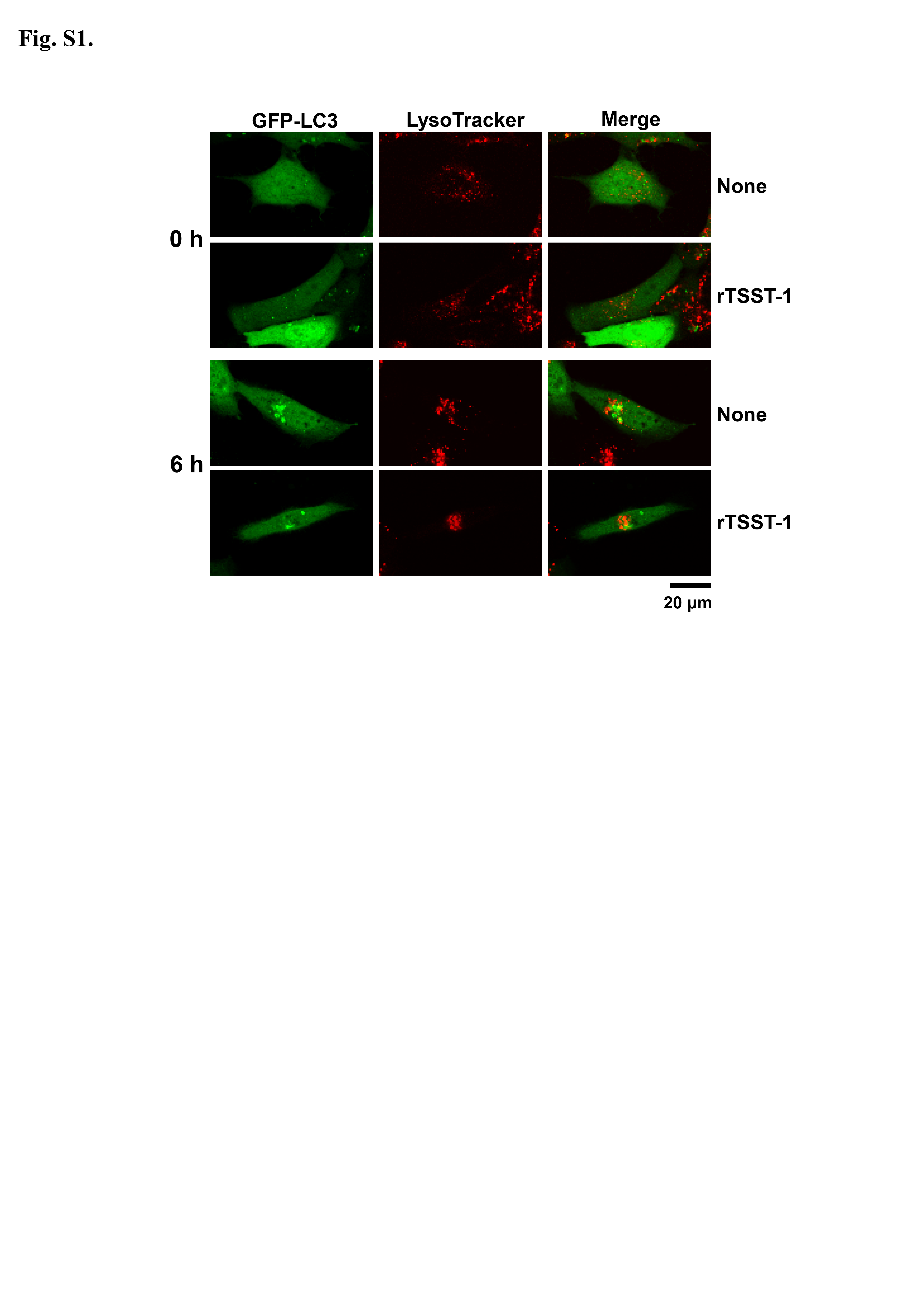

Supplement: Figure S1 — rTSST-1 does not enhance autophagosome and lysosome fusion. (TIF) [file pone.0113018.s001.tif]

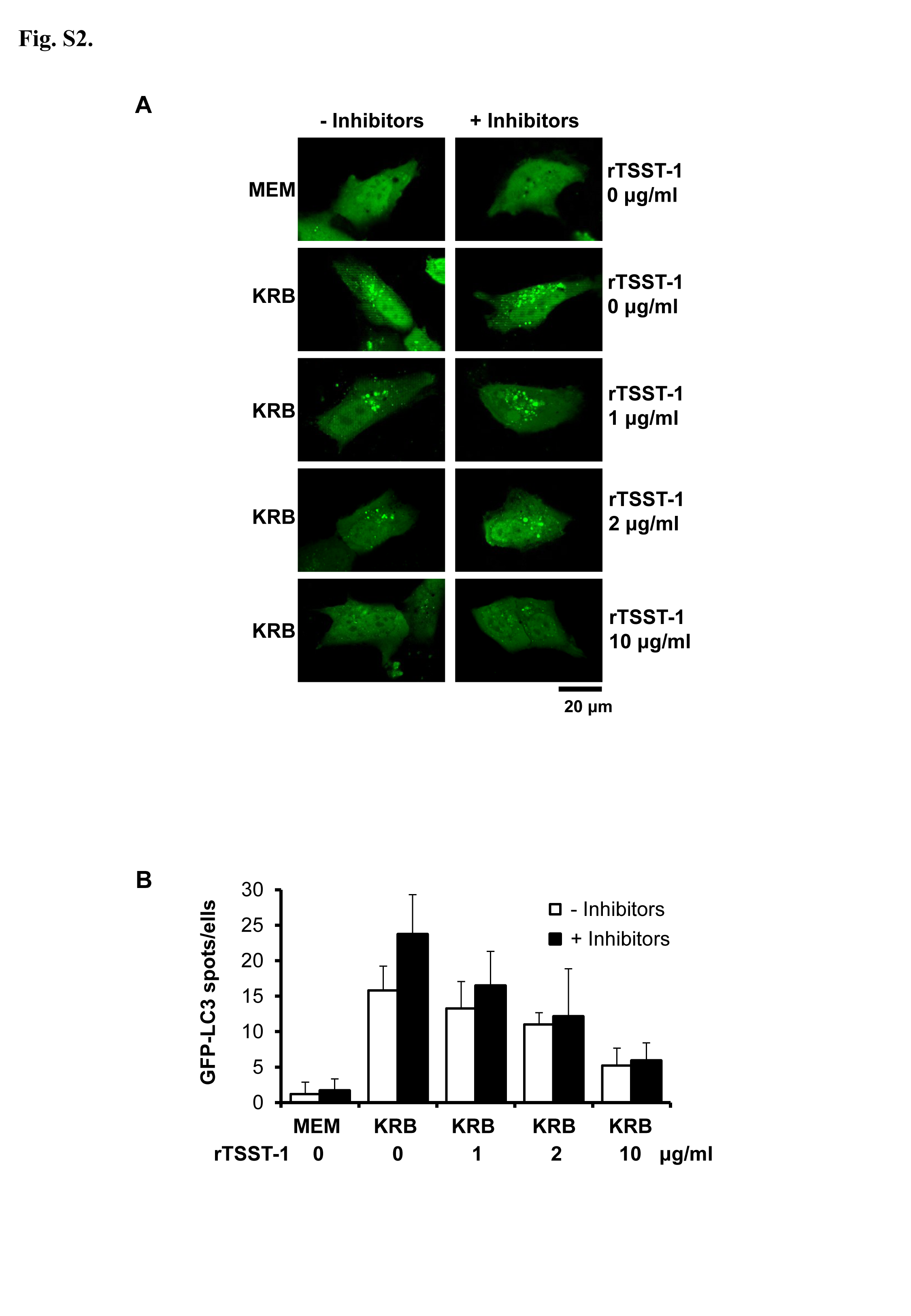

Supplement: Figure S2 — rTSST-1 suppresses GFP-LC3 puncta formation in the nutrient-starved HeLa 229 cells in a dose-dependent manner. (TIF) [file pone.0113018.s002.tif]

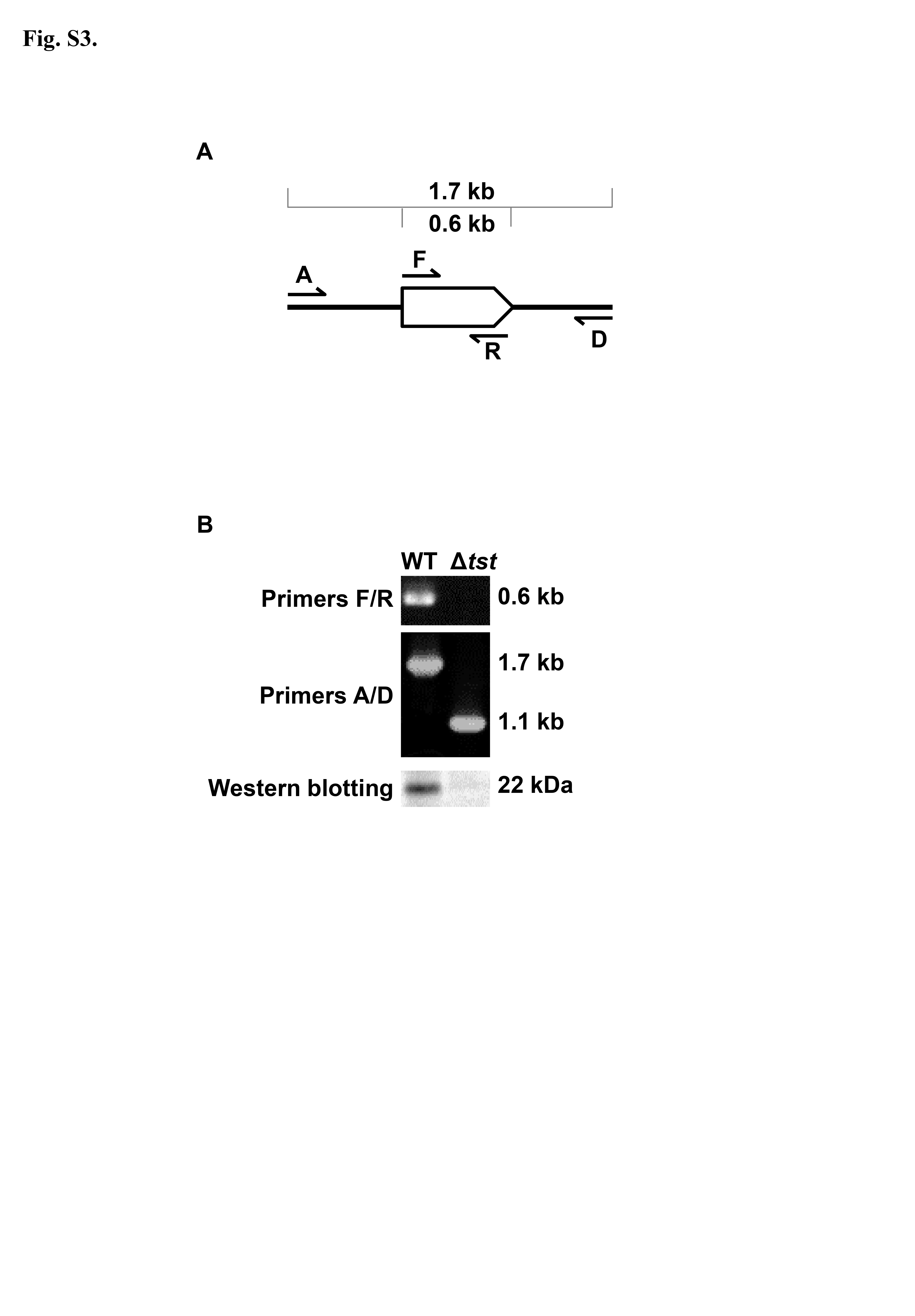

Supplement: Figure S3 — Construction of TSST-1 deficient mutant. (TIF) [file pone.0113018.s003.tif]

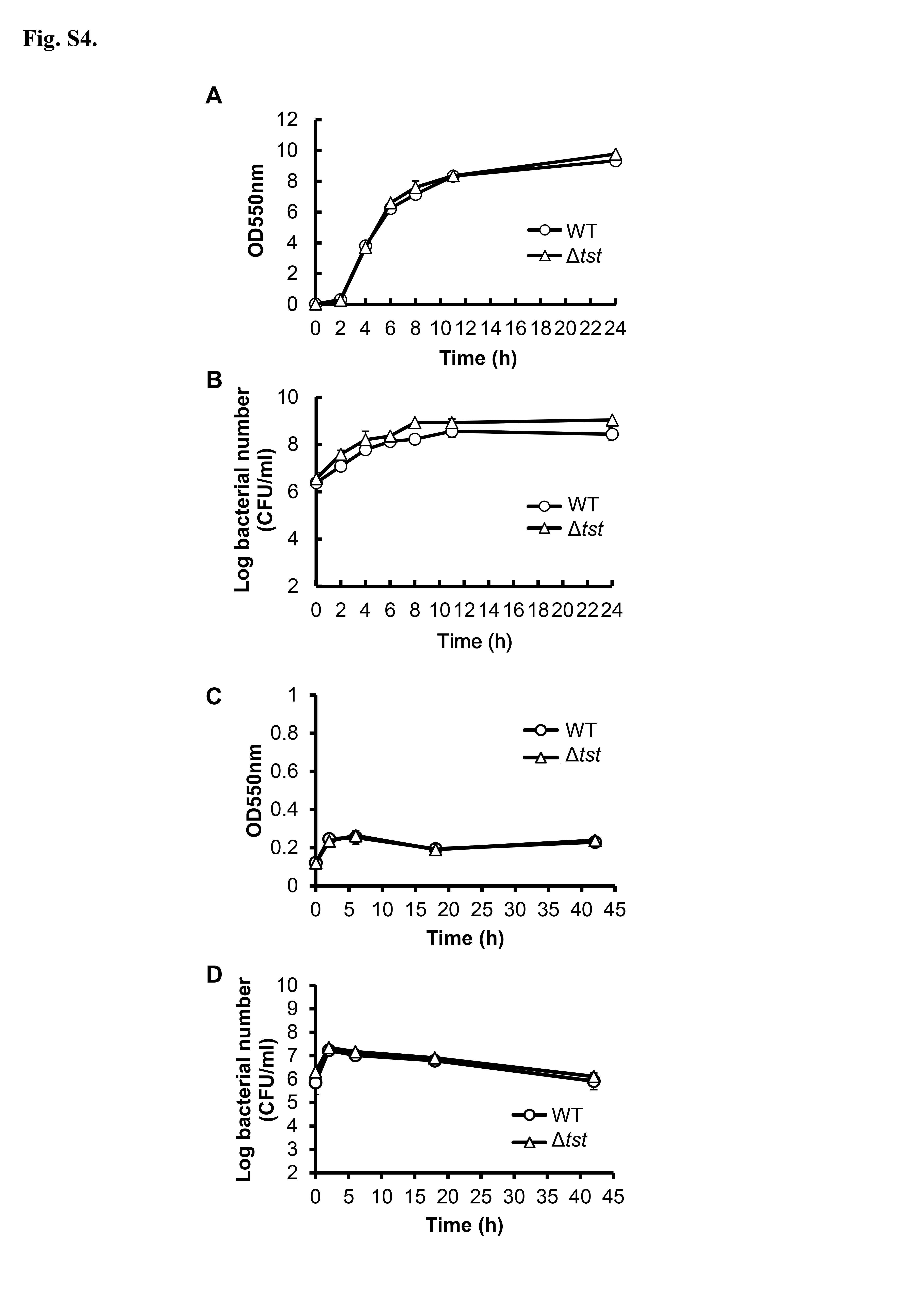

Supplement: Figure S4 — Growth of Δ tst is comparable with that of the WT. (TIF) [file pone.0113018.s004.tif]

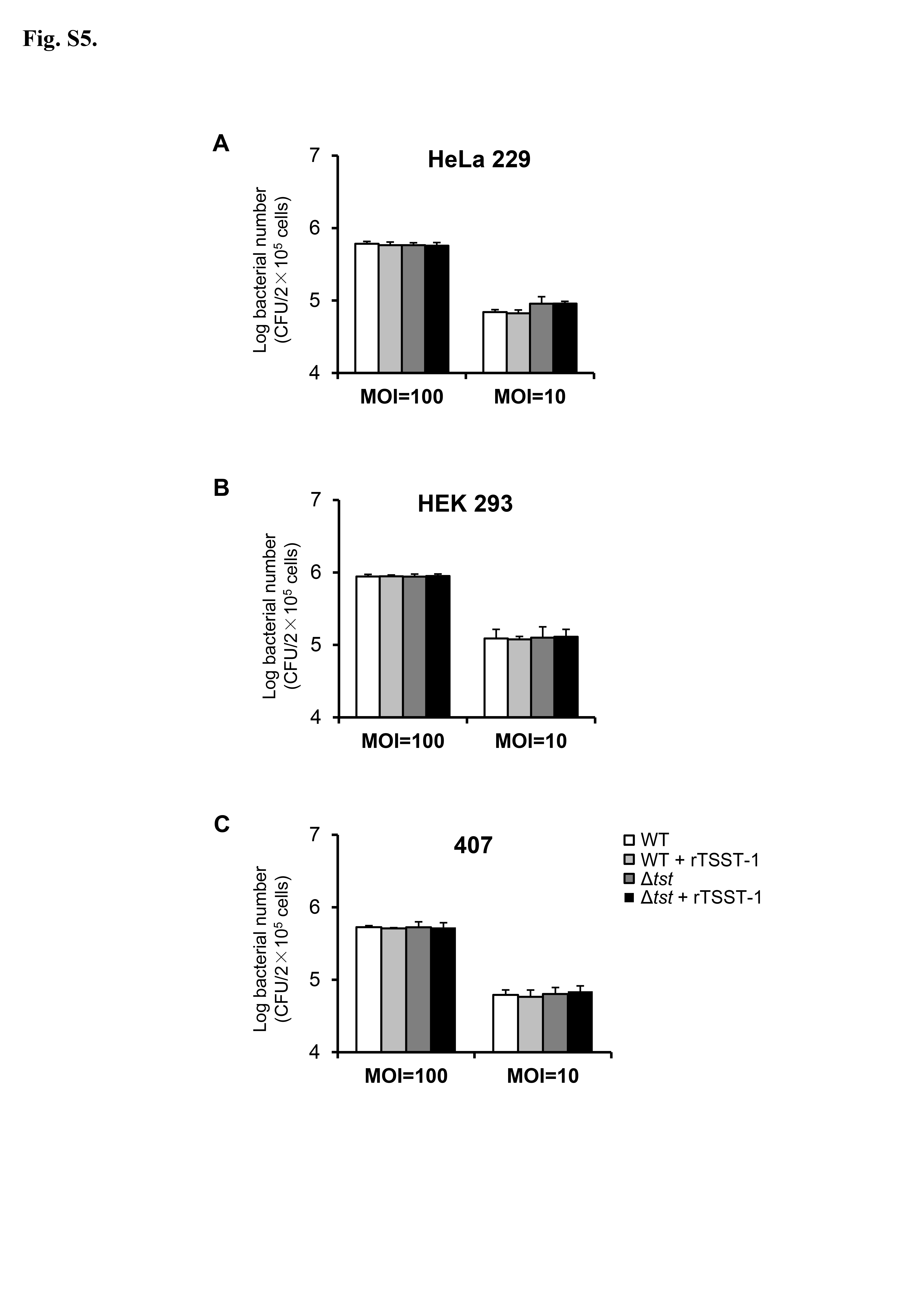

Supplement: Figure S5 — Effect of TSST-1 on adhesion assay. (TIF) [file pone.0113018.s005.tif]

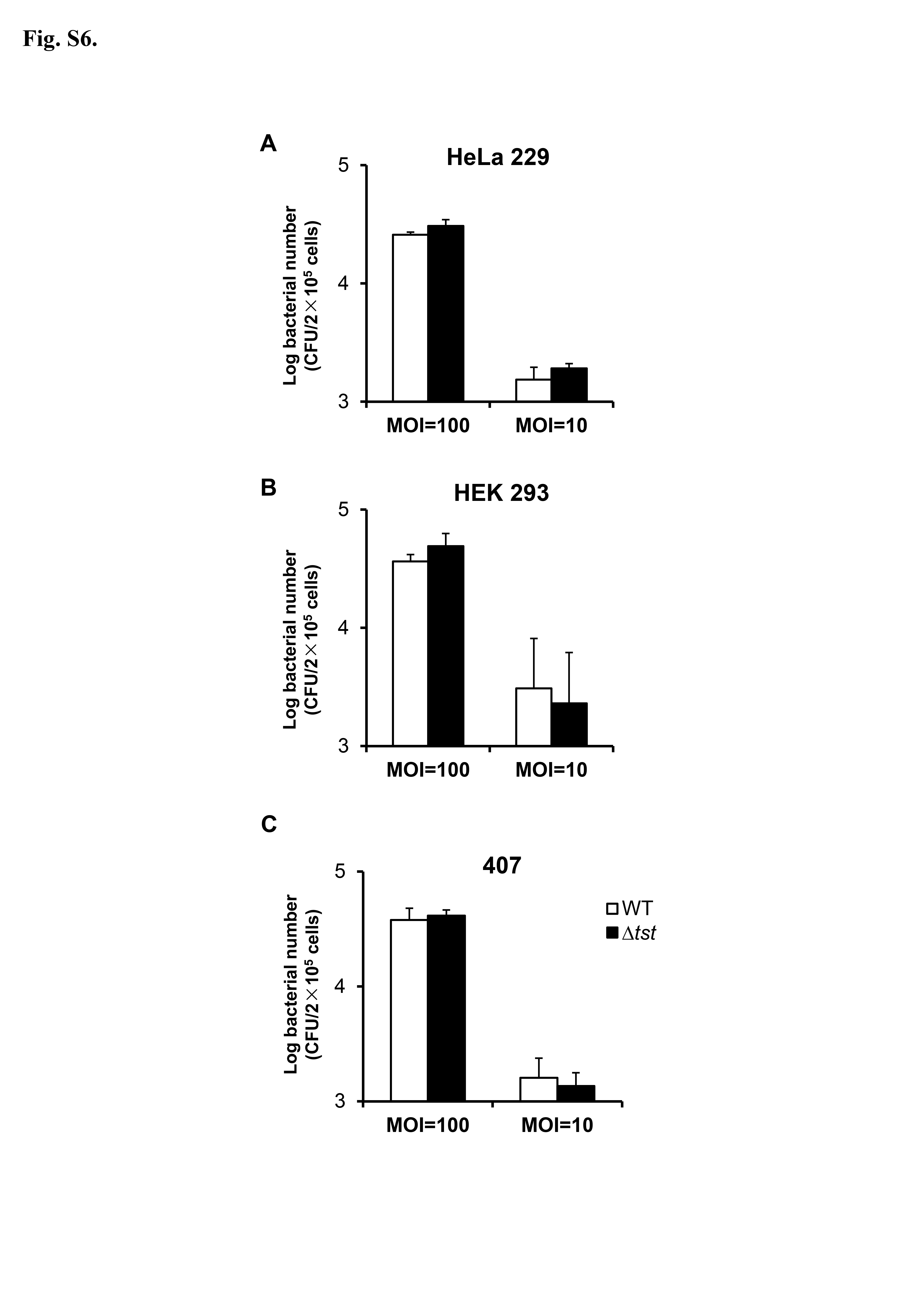

Supplement: Figure S6 — Effect of TSST-1 on invasion assay at MOI 10 and 100. (TIF) [file pone.0113018.s006.tif]

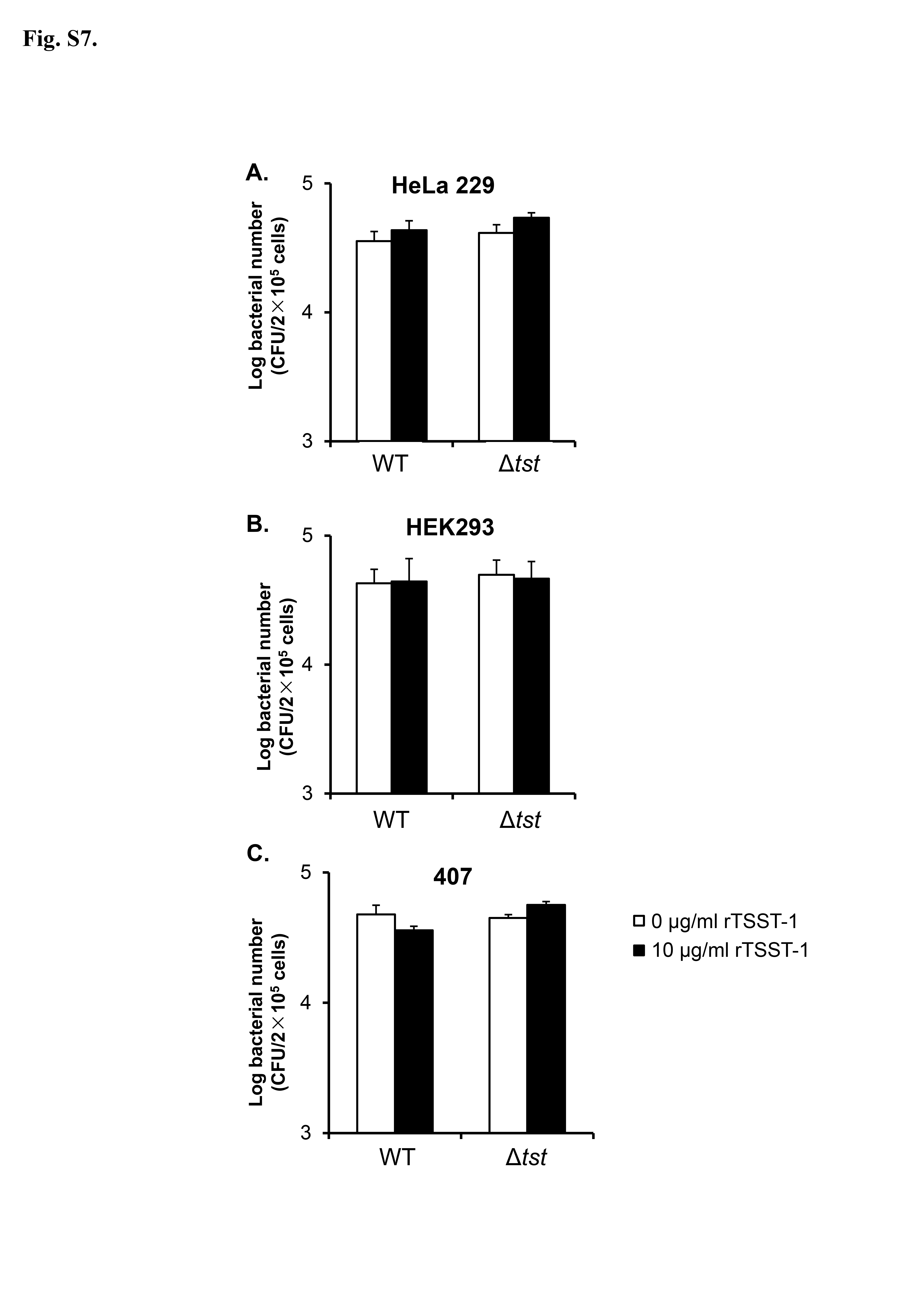

Supplement: Figure S7 — Effect of TSST-1 on invasion assay at MOI 100 in the presence of rTSST-1. (TIF) [file pone.0113018.s007.tif]

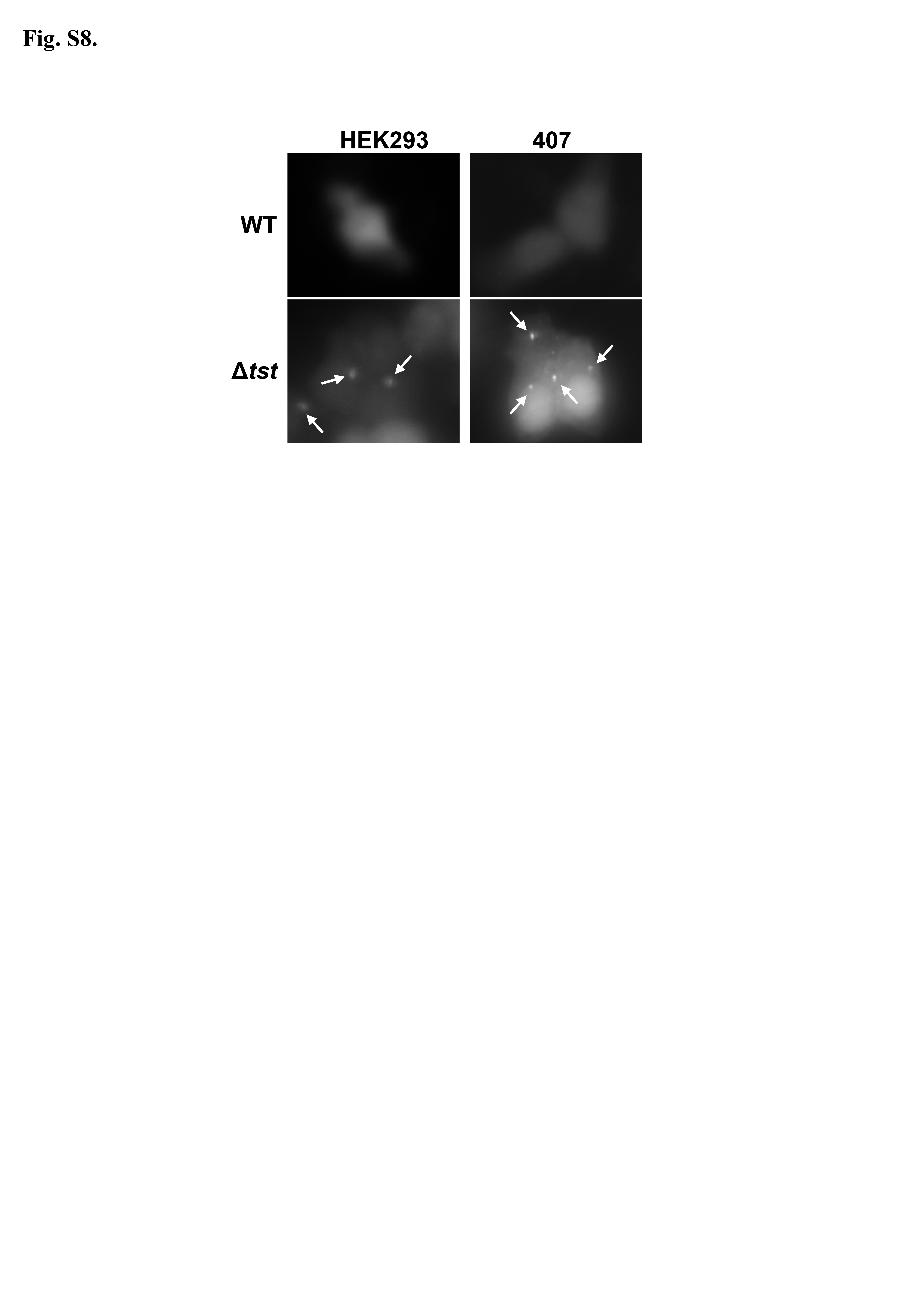

Supplement: Figure S8 — TSST-1-producing S. aureus suppresses autophagy in HEK293 and 407 cells. (TIF) [file pone.0113018.s008.tif]

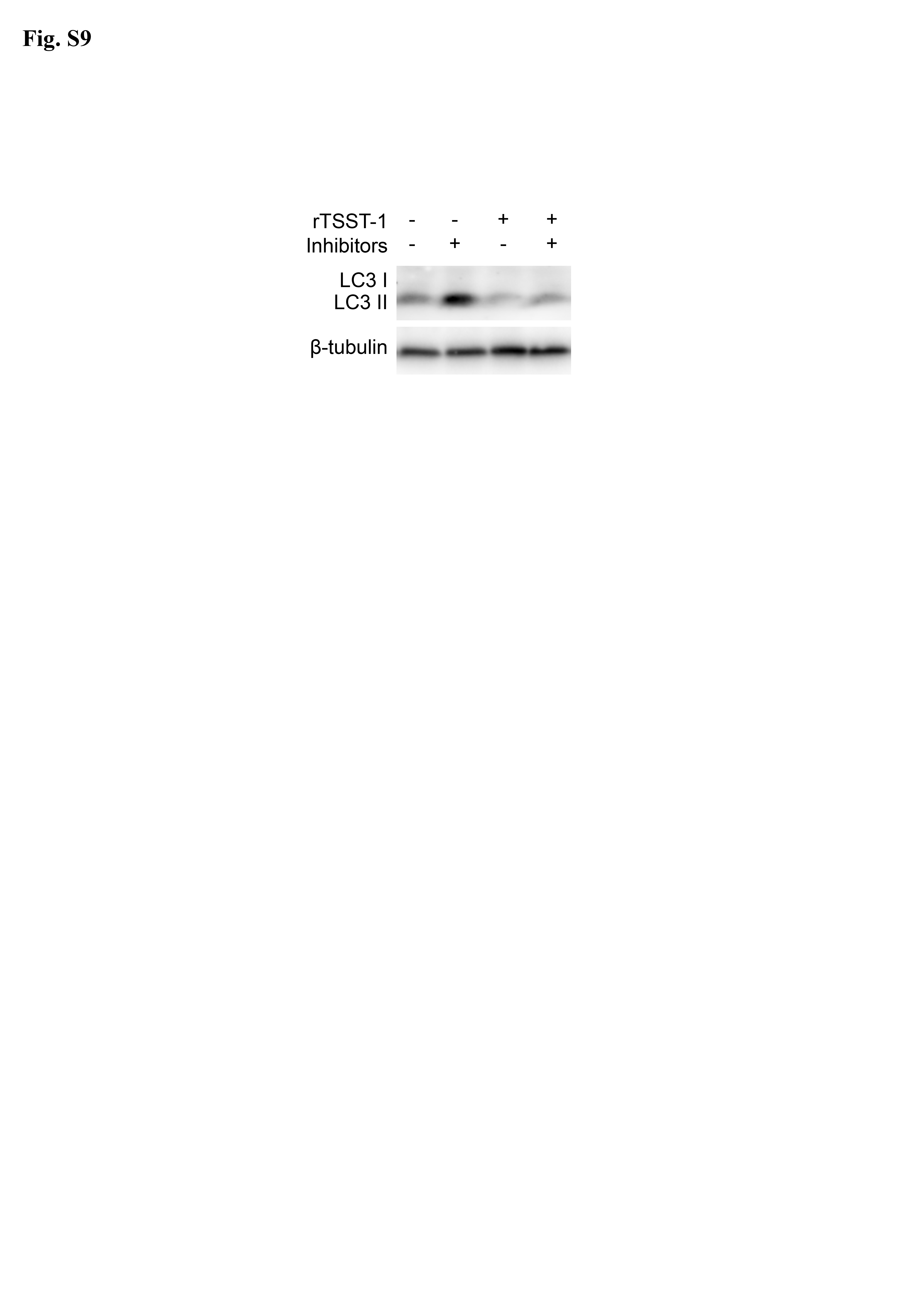

Supplement: Figure S9 — LC3-II accumulation in the Δ tst -infected cells was reduced by addition of rTSST-1. (TIF) [file pone.0113018.s009.tif]
