# Supplementary material for: Suppression of Starvation-Induced Autophagy by Recombinant Toxic Shock Syndrome Toxin-1 in Epithelial Cells
Source: PLoS One. 2014 Nov 17;9(11):e113018. doi: 10.1371/journal.pone.0113018 (PMC4234639; doi:10.1371/journal.pone.0113018)
Supplement: File S1 — Supporting information describing methodologies and results of Lysotracker-stained lysosomes, tst mutant construction and effect of TSST-1 on adhesion and invasion assay of S. aureus . (DOCX) [file pone.0113018.s010.docx]

**Supplementary data**

Materials and Methods

Lysosomal staining by LysoTracker Red DND-99

Autophagy in HeLa 229 cells was induced for 4 h, as described the main text. Lysosomes were stained with LysoTracker Red DND-99 (Invitrogen) according to the manufacturer's protocol. After 30 min of incubation, lysosomes in lived cells were observed under confocal microscopy.

Preparation of rTSST-1, mTSST-1 and SEs

rTSST-1, mTSST-1, SEA, SEB and SEC were prepared as described previously [32,33,34]. Briefly, the *tst*, *sea*, *seb* and *sec2* genes were amplified from *S. aureus* 834, FRI722, 243 and FRI361, respectively. Primer sequences were *tst*/forward; 5’-CCCCGAATTCTCTACAAACGATAATATAAAG, *tst*/reverse; 5’-CACACCCGGGTCGACTTAATTAATTTCTGCTTCTATAG, *sea*/forward; 5’-CCCCGGATCCGAGAAAAGCGAAGAAATAAAT-3’, *sea*/reverse; CCCCGAATTCTGAACATTACGTGTTCAAAACTAC-3’, *seb*/forward; 5’-CCCCGGATCCCAACCAGATCCTAAACCAGATGAGT-3’, *seb*/reverse; 5’-CCCCGAATTCATTTCACTTTTTCTTTGTCGTAAGAT-3’, *sec2*/forward; 5’-CCCCGGATTCGAGAGCCAACCAGACCCTACG, and *sec2*/reverse; 5′-CCCCGAATTCTTATCCATTCTTTGTTGTAAGGTGG. Each gene fragment was inserted into pGEX-6p-1 (Amersham Pharmacia Biotech, Piscataway, NJ), a glutathione-S-transferase (GST) fusion expression vector, and then the plasmid was transferred into *Escherichia coli* DH5α cells. To prepare mTSST-1, histidine at position 135 of the TSST-1 which has been reported to be an important site for superantigenic activity [35] was replaced with alanine by using a selection primer (5’-GCGTGACACCACGATGCCCGCGGCAATGGCAACAACG) and a mutagenic primer (5’-AGACTTTGAAATTCGTGCTCSGCTAACTCAAATAC). These primers were designed to replace the oligonucleotides CAT with GCT at nucleotide number 403 and 405 of the *tst* gene. The plasmid DNA was denatured at 100°C for 5 min in the presence of both the selection and mutagenic primers. The mutant DNA strand was synthesized with T4 DNA polymerase and T4 DNA ligase (Takara, Ohtsu, Japan). The enzymes were inactivated, and the reaction mixture was digested with *Pst*I (Takara). After digestion, an aliquot of the sample was introduced into *E. coli* NM522 mutS. The bacteria were cultured at 37°C overnight in SOC broth (Nissui) supplemented with 100 μg/ml ampicillin. The DNA from the mixed plasmid pool was extracted and digested with *Pst*I again. For the final transformation, an aliquot of the reaction mixture was introduced into *E. coli* DH5α. Several colonies were grown overnight for plasmid DNA extraction, and the plasmid DNA was digested in 2 separate reactions with *Pst*I and *Sac*II (Takara) and analyzed on an agarose gel. Clones that had lost the unique PstI site and acquired an additional *Sac*II site were selected. Then the pGEX-6p-1 containing mutant *tst* gene was transformed into *E. coli* DH5α. Expression of GST-fused rTSST-1, mTSST-1, SEA, SEB and SEC was induced by adding isopropyl-1-thio-β-d-galactopyranoside to a final concentration of 0.5 mM. GST-fusion proteins were purified and GST was removed by using the bulk GST purification modules (Amersham Pharmacia Biotech) according to the manufacturer’s instructions. The purified proteins were quantified by Bradford protein assay (Bio-Rad, Richmond, CA) and analyzed by SDS-PAGE. Superantigenic activity of each protein was confirmed as previously described [32,33,34].

Construction of TSST-1-deficient mutant of *S. aureus* (Δ*tst*)

A temperature sensitive shuttle vector, pAULA [36], was used to construct Δ*tst*. Briefly, 0.6 kb upstream and downstream DNA fragments of the *tst* gene encoding TSST-1 were amplified by PCR using genomic DNA of *S. aureus* 834 as template. The upstream fragment was amplified with primer A: 5’-GAATTCGGTAGGACATTTAATTCTGG-3’ and primer B: 5’-GGTACCAAGCAACAAAGGGCTTACG-3’. The downstream fragment was amplified with primer C: 5’-GGTACCTACAATACTGAAAAACCACC-3’ and primer D: 5’-AAGCTTAAGAAGTTGTGGTCAAACG-3’. The upstream and downstream fragments of the *tst* gene were then combined using *Kpn*I recognition site and the combined product was inserted into pAULA plasmid at *Eco*RI and *Hind*III site. For selection, the tetracycline resistant gene amplified from PHY300PLK plasmid (Takara) using primer P1 (5’-AAGCTTAAGAGTAGTTCAACAAACGG-3’) and primer P2 (5’-AAGCTTCCAAAGTTGATCCCTTAACG-3’) was inserted into the plasmid at *Hind*III site. The plasmid was then electroporated into *S. aureus* 834 at 2000 V/mm with time constant at 5 ms. To achieve allelic exchange, the transformants were cultured at non-permissive temperature [37] and Δ*tst* was selected from tetracycline sensitive clones. Deletion of the *tst* gene was confirmed by PCR using primer A and D and *tst*-specific primers (primer F: 5’-TTGTTGCTTGCGACAATCGC-3’ and primer R: 5’-AATCACTTTGATATGTGGATCC-3’). Lack of TSST-1 production in Δ*tst* was confirmed by Western blotting using anti-TSST-1 antibody produced in our laboratory.

Cell lines and cell culture conditions

Human epithelial kidney HEK293 cells were cultured at 37ºC and 5% CO_2_ in Dulbecco’s modified Eagle medium (Nissui), supplemented with 10% fetal bovine serum (FBS, JRH Biosciences), and 0.03% of L-glutamine (Wako). Human intestinal epithelial 407 cells were cultured in RPMI1640 medium (Nissui), supplemented with 10% FBS, 0.03% of L-glutamine.

Adhesion and invasion assays

HeLa 299, HEK293, and 407 cells were seeded in 24-well plates with 2 × 10^5^ cells/well and incubated with *S. aureus* 834 or Δ*tst* at multiplicity of infection (MOI) of 10 or 100. For adhesion assay, non-adherent bacterial cells were removed after incubation for 15 min by washing with 1 ml PBS for 4 times. For invasion assay, the extracellular bacteria were eliminated after incubation for 45 min with 100 μg/ml lysostaphin (Wako) and the number of intracellular bacteria was determined at 0 h after infection. The bacterial cells were collected in PBS containing 0.5% 3-[(3-Cholamidopropyl)dimethylammonio]-1-propanesulfonate (CHAPS) and enumerated by plate counts on TSA agar plates.

**Results**

rTSST-1 does not enhance lysosomal fusion

To observe effect of rTSST-1 on autophagosome and lysosome fusion, lysosomes in the GFP-LC3-expressing HeLa 229 cells were stained with LysoTracker Red DND-99. The results in Figure S1 indicated that total amount of GFP-LC3 puncta was reduced by the addition of rTSST-1. However, the percent of lysosomes fused with GFP-LC3 puncta did not significantly change or enhance by the addition of rTSST-1.

Construction and characterization of Δ*tst*

To elucidate the role and function of TSST-1 involved in staphylococcal infection in the non-immune cells, Δ*tst* was constructed from the parental strain *S. aureus* 834 via homologous recombination using pAULA-based integration-excision procedure. The deletion of *tst* gene was confirmed by PCR (Figure S3A). By using *tst*-specific primers F and R, 0.6 kb DNA fragment of *tst* gene could not be amplified from the genomic DNA of Δ*tst*. By using primers A and D which anneal upstream and downstream the *tst* gene, 1.1 kb DNA fragment caused by a deletion of 0.6 kb *tst* gene was amplified from the Δ*tst*. Western blotting analysis of secretory proteins revealed that TSST-1 band at 22 kDa found in the WT was absent in the mutant (Figure S3B). Growth curve of this mutant was then observed in comparison to the WT. The results demonstrated that replication capacity of this strain was comparable to the WT in both nutrient rich and nutrient starvation condition (Figure S4).

*TSST-1 has no effect on adhesion and invasion of S. aureus into epithelial cells*

Adhesion and invasion of *S. aureus* WT and Δ*tst* were determined with and without addition of rTSST-1. The results in the Figure S5-S7 indicated that TSST-1 has no significant effect on adhesion and invasion of *S. aureus* into the epithelial cells.

**References**

32. Hu DL, Omoe K, Shimoda Y, Nakane A, Shinagawa K (2003) Induction of emetic response to staphylococcal enterotoxins in the house musk shrew (*Suncus murinus*). Infect Immun 71: 567-570.

33. Hu DL, Omoe K, Narita K, Cui JC, Shinagawa K, et al. (2006) Intranasal vaccination with a double mutant of staphylococcal enterotoxin C provides protection against *Staphylococcus aureus* infection. Microbes Infect 8: 2841-2848.

34. Hu DL, Omoe K, Sashinami H, Shinagawa K, Nakane A (2009) Immunization with a nontoxic mutant of staphylococcal enterotoxin A, SEAD227A, protects against enterotoxin-induced emesis in house musk shrews. J Infect Dis 199: 302-310.

35. Bonventre PF, Heeg H, Edwards CK 3^rd^, Cullen CM (1995) A mutation at histidine residue 135 of toxic shock syndrome toxin yields an immunogenic protein with minimal toxicity. Infect Immun 63: 509-515.

36. Chakraborty T, Leimeister-Wächter M, Domann E, Hartl M, Goebel W, et al. (1992) Coordinate regulation of virulence genes in *Listeria monocytogenes* requires the product of the *prfA* gene. J Bacteriol 174: 568-574.

37. Schäferkordt S, Chakraborty T (1995) Vector plasmid for insertional mutagenesis and directional cloning in *Listeria* spp. Biotechniques 19: 720-722, 724-725.

**Figure legends**

**Figure S1. rTSST-1 does not enhance autophagosome and lysosome fusion.**　HeLa 229 cells were transfected with pEGFP-hLC3. Autophagy was induced under nutrient-starvation condition for 0 and 6 h with or without the addition of 10 μg/ml rTSST-1. Lysosomes were stained with LysoTracker Red DND-99, lysosomes and GFP-LC3 puncta were observed under confocal microscope.

**Figure S2. rTSST-1 suppresses GFP-LC3 puncta formation in the nutrient-starved HeLa 229 cells in a dose-dependent manner.** HeLa 229 cells were transfected with pEGFP-hLC3. Autophagy was induced under nutrient-starvation (KRB) condition with additions of 0, 1, 2 and 10 μg/ml rTSST-1 and lysosomal protease inhibitors. Cells in MEM media with 0 μg/ml rTSST-1 were used as control. At 6 h, GFP-LC3 puncta were assessed under confocal microscope (A). GFP-LC3 puncta were counted from 20 cells of 2 independent-experiments (B).

**Figure S3. Construction of TSST-1 deficient mutant.** (A) Schematic representation of the *tst* gene in the WT, *S. aureus* 834. Annealing positions of primers A, F, R, D are indicated. (B) Gel electrophoresis and Western blotting analysis of Δ*tst* mutant. Lack of *tst* gene in the Δ*tst* was confirmed by PCR using primer pairs, F/R and A/D. The PCR products amplified from the chromosomal DNA of WT and Δ*tst* are shown*.* Lack of TSST-1 in the culture media of the Δ*tst* was confirmed by Western blotting using anti-TSST-1 antibody.

**Figure S4. Growth of Δ*tst* is comparable with that of the WT.** *S. aureus* WT and Δ*tst* were inoculated in brain heart infusion medium (A-B) or KRB buffer (C-D) and cultured at 37ºC under aerobic condition. At indicating time, the optical density of the bacterial suspension was measured at 550 nm (OD550) (A and C) and bacterial cell number was enumerated by plate counts on tryptic soy agar (TSA) plates (B and D). The data are provided as SD of at least 2-independent experiments.

**Figure S5. Effect of TSST-1 on adhesion assay.** (A) HeLa 229, (B) HEK293 and (C) 407 cells were seeded in 24-well plates with 2 × 10^5^ cells/well and incubated with *S. aureus* 834 or Δ*tst* at MOI of 10 or 100 supplemented with or without 10 μg/ml rTSST-1. After incubation for 15 min, non-adherent bacterial cells were removed by washing with PBS. The bacterial cells were collected in PBS containing 0.5% CHAPS and enumerated by plate counts on TSA plates.

**Figure S6.** **Effect of TSST-1 on invasion assay at MOI 10 and 100.** (A) HeLa 229, (B) HEK293 and (C) 407 cells were seeded in 24-well plates with 2 × 10^5^ cells/well and incubated with *S. aureus* 834 or Δ*tst* at MOI of 10 or 100. After incubation for 45 min, the extracellular bacteria were eliminated with 100 μg/ml lysostaphin and the number of intracellular bacteria was determined at 0 h of infection. The bacterial cells were collected in PBS containing 0.5% CHAPS and enumerated by plate counts on TSA plates.

**Figure S7.** **Effect of TSST-1 on invasion assay at MOI 100 in the presence of rTSST-1.** (A) HeLa 229, (B) HEK293 and (C) 407 cells were seeded on 24-well plates with 2 × 10^5^ cells/well and incubated with *S. aureus* 834 or Δ*tst* at MOI of 100 with or without 10 μg/ml rTSST-1. After incubation for 45 min, the extracellular bacteria were eliminated with 100 μg/ml lysostaphin and the number of intracellular bacteria was determined at 0 h of infection. The bacterial cells were collected in PBS containing 0.5% CHAPS and enumerated by plate counts on TSA plates.

**Figure S8.** **TSST-1-producing *S. aureus* suppresses autophagy in HEK293 and 407 cells.** HEK293 and 407 cells were transfected with pEGFP/hLC3 plasmid and infected with *S. aureus* 834 or Δ*tst*. At 6 h of infection, GFP-LC3 was observed under fluorescent microscope (Olympus IX71, Olympus, Tokyo, Japan). Arrows indicate GFP-LC3 puncta.

**Figure S9.** **LC3-II accumulation in the Δ*tst*-infected cells was reduced by addition of rTSST-1.** HeLa 229 cells were infected by Δ*tst* with or without the addition of 10 μg/ml rTSST-1 and lysosomal protease inhibitors. At 6 h of infection, LC3-II was detected by Western blotting. Data are representative of 2-indendent experiments.

**Acknowledgements**

We thank T. Chakraborty, Institute of Medical Microbiology, University Teaching Hospital of Giessen, Germany for providing a thermosensitive shuttle vector, pAULA.
